# Delay discounting of money and health outcomes, and adherence to policy guidelines during the COVID-19 pandemic

**DOI:** 10.3389/fpubh.2022.953743

**Published:** 2022-08-22

**Authors:** Jakub M. Krawiec, Szymon Mizak, Marco Tagliabue, Wojciech Białaszek

**Affiliations:** ^1^Institute of Psychology, Decision Lab: Center for Behavioral Research in Decision Making, SWPS University of Social Sciences and Humanities, Warsaw, Poland; ^2^Department of Behavioural Sciences, OsloMet – Oslo Metropolitan University, Oslo, Norway

**Keywords:** delay discounting, COVID-19, preventive behaviors, adherence, policy

## Abstract

Delay discounting refers to the observation that the subjective value of an outcome decreases as the delay to its receipt increases. It is well-established that steep delay discounting is related to various maladaptive behaviors, including poorer health-related choices. One of the current challenges of public health policies that emerged during the COVID-19 pandemic is to encourage preventive behaviors against infectious diseases. In this study, we aimed to explore possible underpinnings of adherence to COVID-19 related public health policy guidelines such as disinfection, distancing, and masks (DDM). Participants completed monetary and health discounting tasks across two outcome amounts in gain and loss conditions, and they provided self-report measures of adherence to the DDM policy. Contrary to the theoretically plausible prediction that higher discounting rates would be negatively associated with adherence to health-related public policy guidelines, we found no compelling evidence to support such statement. We discuss the potential weaknesses of declarative measurements of attitudes toward COVID-19 and consider using behavioral interventions for influencing discounting rates for complementing and enhancing current policy guidelines.

## Introduction

The COVID-19 pandemic led to tremendous devastation on a global scale. Shortly after the World Health Organization announced the outbreak of the COVID-19 as a pandemic on March 11th, 2020, there have been numerous efforts of cooperation between academicians and policymakers. Especially before vaccines were developed, this cooperation was aimed at preventing the spread of a health threat that has proven to affect people of all ages and across the globe. Since then, policymakers from several countries have been trying to encourage certain types of social behaviors that may prevent or contain the spread of the virus, and these initiatives occur in addition to national and international vaccination campaigns.

It is a relatively common practice to encourage preventive behaviors by using acronyms or simple heuristics to remind people of simple steps to reduce the risk of contracting COVID-19. For example, Japan's principle “Avoid the Three Cs” raises awareness about certain places where it is easier to contract the virus: crowded places, close-contact settings, and confined and enclosed spaces (1). In Poland, these recommended behaviors take the form of simple heuristics that include three rules that residents should keep in mind: disinfection, distancing, and masks (DDM). However, observations at the governmental and societal levels indicate that a significant proportion of the population neither comply with the DDM policy nor intend to get vaccinated (2–5). In this brief research report, we explore how delay discounting may represent one of the possible underpinnings of failing to comply with recommended behaviors that protect against COVID-19 transmission.

Adhering to the DDM policy may be viewed as a set of behaviors that require self-control: choosing a larger but delayed reward over a smaller but sooner, or immediate, reward. Conversely, the opposite set of behaviors may be viewed as impulsive: not disinfecting hands, not practicing distancing, and not wearing a mask can be viewed as strategies that stand against long-term benefits. Delay discounting refers to the phenomenon describing that the present subjective value of a reward declines with delay to its receipt; it is viewed as a mechanism of self-control and impulsivity (6). For example, steep delay discounting is associated with several impulsive maladaptive behaviors such as substance-use disorders (7), cigarette smoking (8), and overeating (9). Since the beginning of the COVID-19 pandemic, several studies have resorted to delay discounting to better understand and explain adherence to preventive behaviors and policy. Examples include exploring the effects of hoarding behavior on a delay discounting task with money and access to surgical masks, which were an especially valued commodity in the early phases of the pandemic (10). In another study, the authors found that framing and reference effects on risk, delay until testing, and positive vaccine safety increased the public's likelihood to practice social distancing (11).

Discounting research often focused on the role of economic rewards or fines due to their universal attractiveness as generalized conditioned reinforcers. However, nonmonetary outcomes are discounted more steeply than money (12), although they may both be described by a similar hyperbolic function, including substance users (13). Also The rates of delay discounting of monetary and nonmonetary outcomes correlate (12), but several studies have demonstrated contrasting delay discounting of monetary and health outcomes (14–16). Moreover, the gain-loss asymmetry, which is also referred to as the sign effect, has been shown to have differential effects not only with monetary outcomes but also with gains and losses of health outcomes [e.g., (17)]. According to the sign effect, gains are discounted more steeply than corresponding losses, including health outcomes, which may be regarded as a bias (i.e., a systematic error) from the normative discounted utility theory (14). Moreover, the sign effect was found to be (weakly) negatively associated with unhealthy outcomes among respondents in Japan (18). According to the magnitude effect, larger rewards tend to be discounted at lower rates and smaller rewards tend to be discounted at higher rates. Rewards may include hypothetical monetary rewards or other commodities [e.g., car rental, see Raineri and Rachlin (19)]. Lastly, according to the domain or commodity effect, the rate of delay discounting may vary depending on the commodity or outcome of a choice. For example, people who have a drug addiction tend to discount hypothetical drug rewards more than hypothetical monetary rewards, but the effects of health and monetary rewards on people who do not have a drug addiction are yet to be soundly demonstrated (20).

Recently Lloyd et al. (21) showed that steeper delay discounting is associated with poorer adherence to social distancing, but not with active violation of lockdown guidelines such as gathering in a group of people. Specifically, those who devalued rewards at higher rates showed lower perceived capability to practice social distancing. Similarly, Byrne et al. (22) found that steeper delay discounting and risky decision-making were associated with lower compliance to mask-wearing and distancing among adults in the USA. In line with these findings, Wismans et al. (23) hypothesized that discounting rates are negatively correlated with declarative measures of adherence to social discounting; however, results revealed opposite relationships. Students who devalued delayed rewards more rapidly declared higher rates of compliance with social distancing and hygiene guidelines. Interestingly, when impulsivity was operationalized as a psychological trait (24), the relationships were as predicted, stating that higher impulsivity correlates negatively with adherence to policy guidelines. In summary, although the current evidence is mixed, discounting processes may represent a valuable conceptual paradigm to study adherence to policies regarding infectious diseases.

The primary aim of this study is to investigate the relationship between respondents' rates of delay discounting of money and health outcomes and their declarations regarding DDM policy guidelines during the COVID-19 pandemic. We were especially interested in the attitudes toward the three DDM preventive behaviors: disinfection, distancing, and (wearing) masks. In Poland, legal authorities drew citizens' attention to these measures for containing the spread of the virus during awareness campaigns. They emphasized DDM as a heuristic that one can easily access and comply to for avoiding the risk of contracting the virus. The secondary aim of this study is to replicate previous findings regarding the similarity of the sign and magnitude effects in the domains of monetary and health outcomes using a brief 5 trial discounting procedure.

## Materials and methods

### Participants

The participants in this study consisted of 515 university students (429 females and 79 males, mean age = 26.82 years; SD = 8.39 years). Consistently with (25), to estimate the sample size for this study we chose an approach comprising a proportion of 20:1 between subjects and the number of items derived from the longest questionnaire (attitudes toward the pandemic: herein not reported) for which we planned to use exploratory factor analysis. Furthermore, the sample size was sufficiently large to detect all anticipated effects. In the end, our final dataset included 338 participants. The data was checked for any failures to meet the attention checks criteria and all participants who did not meet the attention checks' criteria were excluded (see Supplementary materials for a description). The research protocol was approved by the SWPS University Ethics Committee (Faculty of Psychology in Warsaw) and participants gave informed consent before participation. As compensation for their participation, participants were given bonus course credits.

### Materials and procedures

The study was conducted entirely online during the second wave of the COVID-19 pandemic (i.e., December 2020–February 2021) and was addressed to Polish-speaking students at the SWPS University. To explore possible mechanisms of DDM-related decisions, we adopted a well-known discounting instrument: the Five-Trial Adjusting Delay Discounting Task (26) in two domains: monetary and health. The usage of the Five-Trial measure is further supported by the fact that both health and money are discounted hyperbolically (13). Participants made choices between a full amount at different delays and half of the larger delayed consequences, which was available immediately (constantly half of the delayed outcome). In the monetary domain, we used the amounts of 100 and 1,000 Polish zlotys (PLN currency) to be discounted in delay. In the health domain, we asked participants to imagine the prospect of being in excellent health for 4 or 14 days (gain condition) or the prospect of being sick for 4 or 14 days (loss condition).

The DDM measures were constructed as three separate compound variables: attitudes toward disinfection, attitudes toward distancing, and attitudes toward masks. The items used to construct the DDM measures were declarative sentences that required an answer on a Likert scale ranging from 1 to 7, where 1 referred to strong disagreement with the sentence, and 7 referred to strong agreement. The distancing variable included scores from 6 items such as “*I avoid crowded places*”. The disinfection variable included scores from 3 items such as “*I use antibacterial gel or disinfectant wipes*”, and the masks variable included scores from 4 items, such as “*I cover my mouth and nose whenever indoors*”. Compound variables were created by applying factor analysis in R, using the fa function from the psych package (27). Within this method, oblimin rotation was used and the number of factors was set to 1, as separate factor analyses were made for each disinfection, distancing, and masks indices (see Supplementary materials for detailed information). The procedures and measures described in this paper were part of a larger study (see Supplementary materials) that took participants on average 28.15 min (SD = 12.66) to complete.

## Results

Firstly, we replicated the magnitude and sign effects in delay discounting in both domains. These results confirmed that our procedures were valid. The analysis for the magnitude and sign effects were conducted with the use of Bayesian Repeated Measures ANOVA (28). The detailed results may be found in Table 1. Secondly, with the use of Bayesian linear regression, we found evidence that there was no effect of any predictor on adhering to disinfection or distancing recommendations or wearing masks. Bayesian Regression was conducted with the use of the *rstanarm* R package (29).

**Table 1:** *Post-hoc* comparisons for Bayesian RM ANOVA presenting Bayes factors for each comparison.

**Effect**	**Condition**	**Money (BF_10_)**	**Health (BF_10_)**
**Magnitude**	Gains	20.73^(1)*^	43.1^(2)^
(small vs. large amounts)	Losses	0.2^(3)^	0.24^(4)^
**Sign**	Small	1.78*10^∧^30^(5)^	5.76*10^∧^31^(6)^
(gains vs. losses)	Large	2.94*10^∧^16^(7)^	1.87*10^∧^27^(8)^

* For convenience, the number in superscript refers to the order number of the post-hoc comparison.

Using a Bayesian Repeated Measures ANOVA [specifying a multivariate Cauchy prior on the effects (30)], the Bayes factor indicated that the data are 2.095^*^10^54^ times more likely under the model that includes sign and magnitude as the predictors compared to the null model.

In the next step, we tested for differences in discounting between the following conditions: domain (monetary vs. health), magnitude (small vs. large), and sign (gains vs. losses). *Post-hoc* tests between each condition were conducted with the use of the Bayesian *T*-Tests function coming from the *BayesFactor* package (31) in R (32), as JASP does not provide pairwise comparisons returning Bayes Factor for interaction effects. The *post-hoc* tests showed different evidence for alternative hypotheses across conditions. Extremely strong evidence (Bayes Factor over 30) (33), was found in the comparisons between gains and losses of the same magnitude in both domains (comparisons number 5, 6, 7, 8) and in the comparison between small and large gains in the health domain. Moreover, strong evidence was reported for the comparison between small and large gains in the monetary condition (comparison 1), which supports alternative hypotheses. We found no effect in the comparisons between small and large losses in both domains (comparisons 3 and 4).

**Figure 1:**
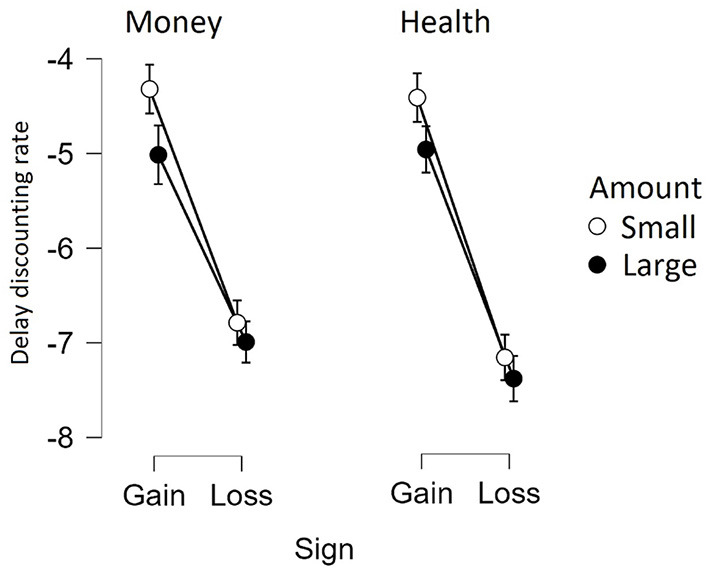
The average discounting rate [ln(*k*)] in gain and loss conditions by outcome magnitude and domain (the whiskers represent 95% credible intervals).

Taken together, our results show extremely strong evidence to support the claim that small monetary and health gains are discounted more steeply than large gains. Furthermore, gains in both domains were discounted more steeply in comparison to losses. The results are illustrated in Figure 1.

In the second stage of our analysis, we investigated whether measures of delay discounting in the health and monetary domains could be potential predictors of behavior consistent with government safety recommendations during the COVID-19 pandemic. A Bayesian linear regression was conducted separately for 3 different dependent variables: attitudes toward disinfection, attitudes toward distancing, and attitudes toward masks. The analysis was done with the use of the *stan_glm* function from *rstanarm* R package (29). The prior distribution was set to normal (0,1) to introduce regularization to account for multiple predictors as a more conservative correction in the case of multiple predictors (34). The fit of the model to the data was performed through visual assessment (35): the observed data was compared to simulated datasets according to the assumed data generating process and the posterior draws of the model parameters. The visual assessment method showed a poor fit of the models to the data (see supplementary information for the Supplementary Figures 1A–C). In other words, the results showed that the observed data were more likely to be generated by the null model compared to the models that included discounting measures. In fact, the results showed strong positive evidence for the absence of any effect of discounting predictors on the DDM measures (all of the credible intervals included 0). Furthermore, as it can be seen from Table 2, the expected value of the parameters is close to zero (mean values).

**Table 2:** Regression coefficients for DDM measures.

**(A) Attitudes toward distancing**
**Domain**	**Sign**	**Amount**	**Mean coefficient**	**Lower CI**	**Higher CI**
Health	gain	large	−2.72e−05	−0.05	0.05
		small	−0.03	−0.08	0.01
	loss	large	−0.01	−0.07	0.05
		small	8.84e−03	−0.04	0.06
Money	gain	large	−0.01	−0.05	0.02
		small	0.03	−0.01	0.08
	loss	large	−0.02	−0.08	0.04
		small	−0.03	−0.09	0.02
**(B) Attitudes toward disinfection**
Health	gain	large	−0.01	−0.06	0.04
		small	−0.01	−0.07	0.04
	loss	large	−0.06	−0.13	0.00
		small	0.04	−0.02	0.1
Money	gain	large	−0.01	−0.05	0.03
		small	0.03	−0.02	0.08
	loss	large	−1.03e−03	−0.07	0.07
		small	−0.02	−0.08	0.04
**(C) Attitudes toward masks**
Health	gain	large	6.65e−03	−0.04	0.06
		small	−0.05	−0.1	0.00
	loss	large	0.02	−0.04	0.08
		small	−4.56e−03	−0.06	0.05
Money	gain	large	−0.02	−0.06	−0.02
		small	0.04	−0.01	0.08
	loss	large	−0.04	−0.1	0.02
		small	−0.06	−0.11	0.00

## Discussion

Public health policy, especially in the COVID-19 pandemic outburst, is one of the most important frontiers and challenges of human existence and wellbeing. One of the promising ways to study adherence to policy guidelines from the perspective of the individual is provided by behavioral economics and research on delay discounting. Steep delay discounting is related to several maladaptive behaviors, ranging from texting while driving to substance abuse, and it has been suggested that it may be a trans-disease process (36, 37). The primary aim of the present study was to investigate the relationship between the rate of delay discounting of monetary and health gains and losses of various magnitudes, and how these may inform adherence to COVID-19-related policy guidelines: specifically, attitudes toward disinfection, distancing, and masks (DDM). Concerning the DDM measures, we did not find any effect of delay discounting on adherence to these guidelines. The secondary aim of this study was to investigate the sign and magnitude effects in delay discounting of monetary and health outcomes. Using a brief discounting procedure in the domain of health outcomes, we replicated robust sign and magnitude effects, which are well established in the literature on discounting of monetary outcomes (14, 38, 39). Although a delay discounting framework is theoretically appealing for explaining possible mechanisms of adherence to the DDM policy, we did not find conclusive evidence of any effect of discounting predictors on these measures. Moreover, our results do not provide sufficient evidence to support the discounting paradigm as a suitable framework for accounting for impulsive behavior in the domain of adherence to COVID-19-related public health policy. Several factors have been found to predict adherence to social distancing and other protective practices against COVID-19. For example, Sadjadi et al. (40) found that communication and support were the strongest enablers of social distancing from an institutional perspective. Without neglecting the interaction of agents and institutions, the temporal discounting perspective of the present study takes a different stance than other psychological and social factors insofar as it considers primarily the relation between the agent and his or her future self. It should be noted that we did not investigate the agents' motives to (fail to) engage in protective behavior: whether with the aim of safeguarding their own health or the health of others. The latter may represent a prosocial purpose and, among others, interpersonal effects have been studied in relation to compliance to social distancing (41). However, the variety of maladaptive behaviors that are associated with delay discounting is mostly related to individual reinforcers that act on the agent, which are theoretically expected to be more apt to being discounted than protective behaviors benefiting others (i.e., discounted less).

Prior research demonstrated that the degree to which future rewards lose value is associated with health-related behaviors such as preventative actions and personal safety (42). On many occasions, individuals who choose better health have to forgo the temptation to choose immediate rewards, and shift their preference toward larger, later outcomes. Concerning adherence to public policy guidelines, which in theory should reflect focusing on larger, later gains, current findings are mixed (21–23). Notoriously, the sources of these discrepancies may be multiple. However, considering that DDM policy guidelines were laid out by the Polish government, one of the variables moderating the relationship between adherence to governmental guidelines and impulsivity might be related to an individual's trust in government. Specifically, the perception of policy-makers as untrustworthy may contribute to reduced compliance with provided rules (41, 42).

Our methodology for measuring discounting rates in a form of a 5-trial discounting procedure can be regarded as valid. In the domain of monetary outcomes, we replicated previous findings, indicating steeper discounting of gains as opposed to losses, and the presence of a magnitude effect for gains. The same pattern was observed concerning the discounting of health outcomes. However, one of the limiting factors in our study was a declarative and subjective measurement of the adherence to the DDM policy. The participants in our study indicated on a 7-point Likert scale (ranging from 1—never or almost never to 7—always or almost always) “*How often do you adhere to the following behaviors to protect yourself from being infected with the coronavirus?*”. Conversely, Byrne et al. (22) asked participants about social discounting in the form of a specific count measure related to the number of people they met without wearing masks or practicing social discounting; furthermore, they asked how many times participants engaged in various behaviors related to social discounting. Thus, one limitation of this study includes a subjective rating on a Likert scale that involves some degree of variability from respondent to respondent. For example, it has been shown that steeper delay discounting was related to poorer compliance with medical guidelines (measured by pill count), but not to self-reported adherence measures (43). Another limitation concerns our sample: it was not a representative sample and caution should be used when extending our results to a general population. Additionally, it has been previously shown that various cultural, socioeconomic, and psychological variables may moderate the relationship between intertemporal choice and constructs of interest (44–46). Although there could be cultural differences that contribute to our null findings, we do not know how our sample reflects any specificity of Polish culture. Cross-cultural comparisons would be beneficial in future studies. Delay discounting may be influenced by cultural traits (47) but some of these differences may stem from economic inequalities and general financial circumstances, rather than culture-specificity (48).

We can imagine multiple ways to change individual behavior. In terms of public health policies, the main aim is to modify behavior in a given population. Recently, one of the promising ways to impact policy-related decisions is through behavioral interventions, specifically nudges, and boosts (49). Nudges refer to contextual changes as part of public policy interventions meant to promote a particular choice (50). Nudges should be considered complements to traditional policy tools rather than substitutes for them (51). Although such direct interventions facilitate behavioral change (52), they are not always effective. For example, introducing messages appealing to social norms on fliers did not seem to enhance the level of understanding of how they may best respond to the COVID-19 pandemic according to recommendations from the government (53). Although we found no support that delay discounting processes may be related to complying with security behaviors, it seems worth exploring any indirect behavioral interventions. It has been previously shown that various manipulations impact delay discounting (54). For example, Kang and Ikeda (18) suggested resorting to nudges and other direct intervention policy tools for counteracting the impulsive and unhealthy practices enacted by naïve delay discounters who are not aware of their future self-control issues. Conversely, sophisticated delay discounters do not seem to need this additional policy intervention. This is one of the features underlying nudging as an exercise of asymmetric paternalism from the policymaker's perspective: “*it creates large benefits for those who make errors, while imposing little or no harm on those who are fully rational*” (55). If there had been enough evidence that the rate of devaluation of future outcomes can be viewed as a mechanism of important behaviors from the perspective of general guidelines or policy, it might be valuable to affect the rate of delay discounting; therefore, a possible cause of impulsive behavior. Another promising way to affect impulsivity is through episodic future thinking (EFT) (56, 57). It has recently been demonstrated that EFT promotes not only far-sighted decisions, but it also increases intentions to engage in preventive behaviors during the COVID-19 pandemic (58).

Although nudging and boosting may be effective at establishing new behavioral repertoires that may commit policy recipients to a better course of action, for example through DDM policy guidelines, the functional relation with and role of the consequences of any (preventive) behavior are key for maintaining and transmitting further the repertoire. Thus, policymakers should analyze these contingencies and design interventions that take into account setting stimuli and both immediate and delayed consequences of preventive behaviors if they are to successfully predict, control, and influence it; possibly, in a lasting way. This seems particularly relevant in the context of the COVID-19 pandemic, as behavioral modification has led to changes in our cultural practices (e.g., physical proximity, home-work, global travel, face mask use) [see also Couto et al. (59)].

## Data availability statement

The raw data supporting the conclusions of this article will be made available by the authors, without undue reservation.

## Ethics statement

The studies involving human participants were reviewed and approved by Komisja ds. Etyki Badań Naukowych Wydziału Psychologii w Warszawie, SWPS University of Social Sciences and Humanities. The participants provided their written informed consent to participate in this study.

## Author contributions

JK: conceptualization, data curation, formal analysis, investigation, methodology, resources, software, validation, visualization, writing—original draft, and writing–review and editing. SM: conceptualization, formal analysis, investigation, methodology, resources, software, validation, and writing—review and editing. MT: writing—original draft and writing—review and editing. WB: conceptualization, funding acquisition, methodology, project administration, resources, supervision, validation, writing—original draft, and writing—review and editing. All authors contributed to the presented work. Authors took part in drafting or revising it critically for important intellectual content and approved the final version to be published. Also, all authors ensured that questions related to the accuracy or integrity of any part of the work are appropriately investigated and resolved.

## Funding

Article Processing Charges (APC) for this article were financed by research subsidies from SWPS University (Project Number 22/2022/FRBN/D).

## Conflict of interest

The authors declare that the research was conducted in the absence of any commercial or financial relationships that could be construed as a potential conflict of interest.

## Publisher's note

All claims expressed in this article are solely those of the authors and do not necessarily represent those of their affiliated organizations, or those of the publisher, the editors and the reviewers. Any product that may be evaluated in this article, or claim that may be made by its manufacturer, is not guaranteed or endorsed by the publisher.
