# Supplementary material for: Delay discounting of money and health outcomes, and adherence to policy guidelines during the COVID-19 pandemic
Source: Front Public Health. 2022 Aug 22;10:953743. doi: 10.3389/fpubh.2022.953743 (PMC9441622; doi:10.3389/fpubh.2022.953743)
Supplement: Supplementary file 1 [file Data_Sheet_1.pdf]

Supplementary Material

1 Procedure, materials, and rationale for reported analyses

As part of the study, each participant also completed a set of questionnaires consisting of Positive and Negative Affect Schedule (Crawford & Henry, 2004; we adapted the PANAS test to include an additional scale measuring affect related to pandemic), State-Trait Anxiety Inventory (Spielberger, 1983), Acceptance and Action Questionnaire (AAQ-II; Bond et al., 2011), and a set of scales measuring attitudes towards sanitary restrictions and vaccinations, including attitudes towards disinfection, distancing, and masks (DDM) measures. Furthermore, we used a 5-trial discounting procedure for probability discounting, but it was not included in the analyses.

Analyses reported in the current study did not include any tasks or measures of probability discounting and focused instead on the effects of delay on choices. Naturally, COVID-19 transmission in society is the result of a series of probabilistic events, and so is the future, as it bears a degree of inherent risk. Our rationale to focus on delay discounting was dictated by the fact that in our opinion adhering to the DDM policy may be viewed as a set of behaviors that require self-control: choosing a larger but delayed consequence over a smaller but sooner, or immediate, reward. Conversely, the opposite set of behaviors may be viewed as impulsive: not disinfecting hands, not practicing distancing, and not wearing a mask can be viewed as simpler strategies that stand against long-term benefits. However, the effects of the virus from the agent's perspective seem less probabilistic and rather a function of several characteristics that could not be accounted for in a standardized procedure (e.g., developing symptoms based on vaccination status, underlying medical conditions, age, gender, etc.). Moreover, the relatively short-term effects of COVID -19 in terms of incubation and lengths of symptoms (excluding the so-called long COVID, currently under investigation) make it more difficult to apply a probability discounting perspective as in other medical areas, such as smokers developing lung cancer over longer delays and with increasing probability.

1.1 Order of tasks

The order of tasks was quasi-random with predefined groups of conditions. Main blocks, that were presented in random order were as follows: 1) large delayed or probabilistic monetary gains and losses, 2) small delayed or probabilistic monetary gains and losses, 3) large delayed or probabilistic health gains and losses, 4) small delayed or probabilistic health gains and losses. Within each block, the order of each condition (gain/loss*delay/probability) was quasi-randomly presented. Assessment of other variables was done in separate, standalone blocks, which were presented in a random order, mixed among main blocks described above. We introduced this presentation of different conditions by blocking them to make it easier to follow for participants (i.e. within one randomly selected block, participants completed tasks referring to gains and losses of a given amount of commodity.

1.2 Construction of DDM measures

Compound variables were created by applying factor analysis in RStudio, using the fa function from the psych package (Revelle, 2022). The chosen method for conducting factor analysis was "principal axis method". Within this method, oblimin rotation was used, and the number of factors was set to 1 as separate factor analyses were made for each DDM index. This way of creating indicators was chosen as a better way than simply calculating the mean or the sum of item scores. To create each indicator, we followed theoretical assumptions – as a base of each factor, items relating to particular kind of protective behaviors were chosen. We present all items with their factor loadings in brackets below. The items were answered with the use of a Likert scale, wherein 1 referred to strong disagreement and 7 referred to strong agreement with the statement:

a) Attitudes towards wearing **masks** included scores from 4 items.
   i) I cover my mouth and nose when outdoors (0.594)
   ii) I cover my mouth and nose indoors (0.535)
   iii) Is the obligation to use face masks outdoors justified? (0.696)
   iv) Is the obligation to use face masks indoors justified? (0.724)
b) Attitudes towards **disinfection** included scores from 3 items
   i) Is it reasonable to wash your hands frequently or disinfect them with alcohol-based liquids? (0.544)
   ii) I wash my hands regularly (0.713)
   iii) I use disinfectant gels or wipes (0.739)
c) Attitudes towards **distancing** included scores from 6 items
   i) I keep a distance of 1.5m from people in public spaces (0.739)
   ii) I avoid crowded places (0.764)
   iii) If I can, I limit my trips out of the house (0.620)
   iv) Is it reasonable to prohibit meetings and events of any kind (0.690)
   v) Is it reasonable to maintain a minimum distance of 1.5m between pedestrians (0.786)
   vi) Is it reasonable to recommend avoiding crowded places (0.721)

Supplementary Table 1. Descriptive statistics for discounting and DDM measures

**A) Discounting measures**							
**Domain**	**Sign**	**Amount**	**Mean**	**Standard deviation**	**Min**	**Max**	**Kurtosis**
Health	gain	large	-4.98	3.29	-9.12	3.18	-0.54
		small	-4.43	3.38	-9.12	3.18	-0.73
	loss	large	-7.40	2.44	-9.12	3.18	2.58
		small	-7.18	2.64	-9.12	3.18	0.92
Money	gain	large	-5.01	3.57	-9.12	3.18	0.60
		small	-4.32	2.91	-9.12	3.18	1.22
	loss	large	-6.99	2.24	-9.12	3.18	2.77
		small	-6.79	2.52	-9.12	3.18	2.24
**B) DDM measures**							
Disinfection		-	0.00	1.17	-4.37	1.21	-0.01
Distancing		-	0.00	1.07	-3.72	1.39	0.66
Masks		-	0.00	1.16	-4.56	1.37	0.76

2 Data exclusion

In the final dataset, we included only participants who completed the entire procedure. Additionally, attention checks were used for data exclusion. Subjects who did not meet at least 2 attention checks were excluded from the analyses. Attention checks were items that demanded participants to read carefully the question and select the most logical or common sense answer. These items were:

a) Which of the following statements is not true
   i) We have four seasons
   ii) The sun revolves around the earth
   iii) A day has twenty-four hours
   iv) Seventeen is higher than twelve
b) What is two times ten minus two?
   i) 18
   ii) 16
   iii) 20
   iv) 24
c) What do you choose?
   i) 0 PLN now
   ii) 1000 PLN in one year
   iii) 1000 PLN in 78 days
   iv) 1000 PLN now

Furthermore, participants whose time of completing the study was shorter than 13 minutes or longer than 90 minutes were excluded from the dataset. This range of time was chosen during pretesting and later, during careful examination of the dataset - we could see that participants who performed quicker or longer than the mentioned time range, were either clicking without consideration nor reading carefully (shorter than 13 minutes) or approaching the procedure multiple times (longer than 90 minutes). One of the reasons for participants' inattention or nonchalance could be the length of the procedure and the online form of it - we suppose that the long procedure might appear mundane for students. Participants whose answers contained inadequate or mocking information were also excluded (e.g., the helicopter model given as gender).

3 Supplementary Figures

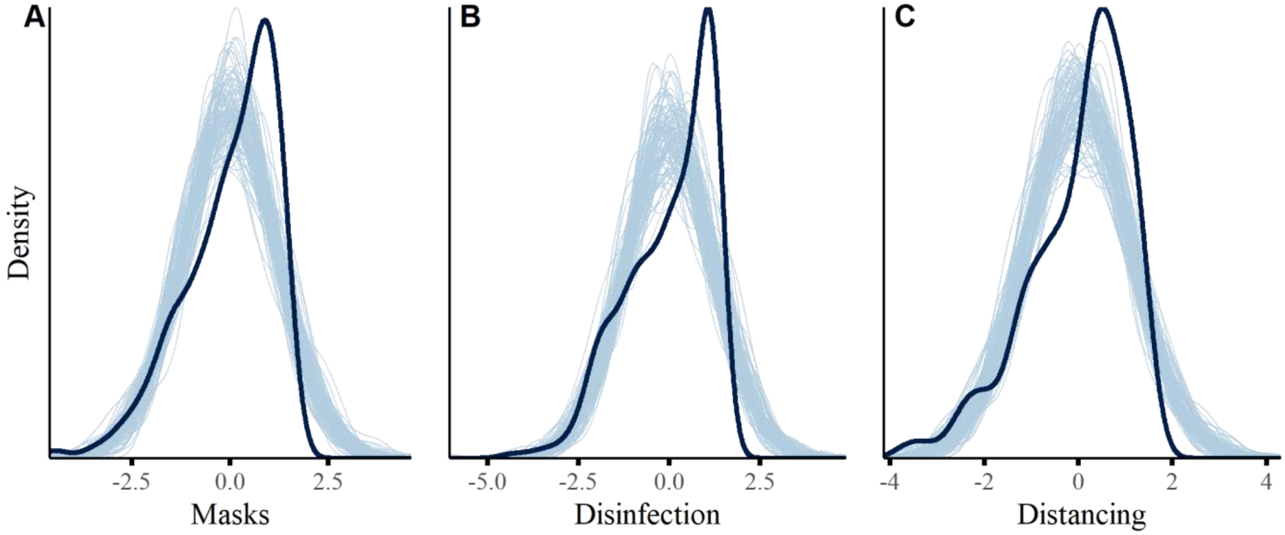

Supplementary Figure 1. The comparison of posterior predictive distribution to observed data for: A) attitudes towards wearing masks, B) attitudes towards disinfection, C) attitudes towards distancing.
